# Comparison of Environmentally Friendly Cleaning Agents and Organic Solvent Cleaning Processes in the Fabrication of Flexible Nine-in-One Microsensors and Their Application in Hydrogen/Vanadium Redox Flow Batteries

**DOI:** 10.3390/mi16111219

**Published:** 2025-10-26

**Authors:** Chi-Yuan Lee, Guo-Bin Jung, Huan-Chu Chen, Mau-Hsiung Chen, Chia-Hung Chen, Kuan-Ting Lai, Cheng-Kai Liao, Yung-Lin Chang, Hao-Peng Chang

**Affiliations:** 1 Department of Mechanical Engineering, Yuan Ze Fuel Cell Center, Yuan Ze University, Taoyuan 32003, Taiwan; guobin@saturn.yzu.edu.tw (G.-B.J.); chenjahon@gmail.com (C.-H.C.); abc0982587414@gmail.com (K.-T.L.); kane025897@gmail.com (C.-K.L.); sky0908865306@gmail.com (Y.-L.C.); 47077paul1951@gmail.com (H.-P.C.); 2 Sea Energe Environmental Industry, Co., Ltd., Taoyuan 32003, Taiwan; se-eusa@seaenerge.com.tw (H.-C.C.); seaenerge.sales@gmail.com (M.-H.C.)

**Keywords:** MEMS, flexible nine-in-one microsensor, HVRFB, cleaning agent, traditional organic solutions (acetone; methanol; and isopropyl alcohol), PI substrate, process compatibility, green process, multi-parameter monitoring

## Abstract

This study focuses on the pre-treatment cleaning technology for the polyimide (PI) substrate of flexible nine-in-one microsensors. The environmentally friendly cleaning agent B, developed by Sea Energe, was innovatively used to replace traditional organic solutions (acetone, methanol, and isopropyl alcohol) to verify its feasibility and application potential in Micro-Electro-Mechanical Systems (MEMS) processes. Cleaning agent B, developed by Sea Energe, was used for the first time to clean the PI substrate of flexible nine-in-one microsensors, and the flexible nine-in-one microsensor was used as a verification platform to compare the cleaning performance with traditional organic solutions (acetone, methanol, and isopropyl alcohol). The experimental results proved that cleaning agent B developed by Sea Energe effectively removed contamination from the PI substrate surface while avoiding the environmental impact and process compatibility issues associated with traditional organic solvents. To verify its reliability, the developed flexible nine-in-one microsensor was embedded in the hydrogen end flow channel of a hydrogen/vanadium redox flow battery (HVRFB) to perform real-time monitoring of multiple parameters, including hydrogen concentration, voltage, current, conductivity, temperature, humidity, flow, pressure, and pH. The experimental results proved that using cleaning agent B, developed by Sea Energe, to clean the PI substrate and the subsequent flexible nine-in-one microsensor resulted in comparable operational stability and measurement accuracy to traditional organic solution (acetone, methanol, and isopropyl alcohol) cleaning processes. This experimental result verifies that cleaning agent B, developed by Sea Energe, not only has an excellent cleaning effect, but also meets the requirements for highly reliable microsensor development, potentially offering an alternative solution for the future introduction of green processes into semiconductors, MEMSs, and various application fields.

## 1. Introduction

In order to accelerate global climate governance, COP29 proposed specific strategies in 2024, including expanding the scale of renewable energy construction, upgrading existing power grids and energy storage infrastructure, and for the first time, incorporating low-carbon hydrogen energy into the core technology field of energy transformation [1]. Similarly, Taiwan also followed the international trend of carbon reduction and promulgated the Climate Change Response Act in 2023. Taiwan clearly stipulates that “net zero emissions by 2050” is the long-term development blueprint and promotes energy transformation simultaneously through policy tools such as renewable energy promotion, a carbon pricing system, and energy storage technology integration [2]. Statistics show that Taiwan’s total greenhouse gas emissions in 2022 decreased by 4.07% compared with the previous year, indicating that the relevant carbon reduction policies have shown preliminary results [3]. Finally, under the dual challenges of climate deterioration and energy security, Taiwan can simultaneously achieve multiple goals such as stable power supply, carbon neutrality, and sustainable development in the process of energy transformation only by accelerating low-carbon emissions of energy systems and actively introducing key technologies such as hydrogen energy, long-duration energy storage, and smart grids [4].

With the rapid growth of the global population, industrialization, and increased mobility of people, stable power supplies and sustainable development have become core challenges that society must face today. As the population continues to expand, land use patterns have changed significantly, and with the increase in greenhouse gas emissions, air quality has deteriorated and long-term damage has been caused to the ecosystem [5]. Energy consumption has increased year by year, especially the high dependence on fossil fuels, which has become the main source of large-scale emissions of carbon dioxide and other air pollutants. Although renewable energy sources, such as solar energy and wind energy, have the potential to reduce carbon emissions, they are intermittent and volatile due to natural conditions, making it difficult for power supply to meet actual demand in a timely manner [6]. Therefore, in order to effectively fill the gap between energy supply and demand and maintain the stability of the power grid, the development of efficient and scalable energy storage technology has been widely regarded as a key strategy to promote energy transformation and achieve carbon neutrality goals.

In semiconductor and Micro-Electro-Mechanical Systems (MEMS) manufacturing, volatile organic solvents such as acetone, methanol, and isopropyl alcohol (IPA) are widely used for substrate and wafer cleaning; however, they are also among the major sources of air pollution and greenhouse gas emissions. Hsu et al. reported that cleaning operations in Taiwanese semiconductor fabs emit approximately 0.038 ± 0.016 kg of VOCs per liter of solvent used, with IPA being the dominant species [7]. Globally, VOC emissions from the electronics manufacturing industry account for about 15% of total industrial VOC emissions, posing significant environmental and occupational health challenges [8]. Although green cleaning technologies such as supercritical CO_2_ and plasma-based cleaning have demonstrated environmental potential, they remain limited by high equipment cost, complex operation, and poor compatibility with polymeric substrates such as polyimide (PI) [9]. Therefore, the development of a VOC-free, low-energy, and process-compatible cleaning process has become a crucial direction for achieving sustainable semiconductor manufacturing.

The HVRFB [10] is an emerging long-duration energy storage technology that combines the advantages of high efficiency, long cycle life, high safety, and high-power output. In this research design, the anode reaction is converted into a hydrogen evolution reaction to replace the structure in the traditional VRFB that both positive and negative electrodes rely on for vanadium redox coupling. Its core reaction mechanism is as follows: the cathode end maintains the common VO^2+^/VO_2_^+^ redox reaction of the VRFB, while the anode end uses hydrogen as the reactant, releasing protons and electrons through the hydrogen oxidation reaction, and transporting the electrons to the external circuit to generate electricity. Since it is no longer limited by the multivalent state conversion of vanadium at the anode end, the anode design of the HVRFB is more flexible, which can simplify the system’s configuration and improve the stability of the electrochemical reaction, ultimately helping to improve overall system operating efficiency. In addition, vanadium ion crossover is a key issue affecting the long-term stability of HVRFBs, as it may lead to electrolyte imbalance and monitoring errors. Although this study mainly focuses on the sensor cleaning process, optimizing the internal environment of the battery is crucial for ensuring the reliability of the sensing data.

Meanwhile, hydrogen energy and redox flow batteries have been widely regarded as key technologies for next-generation long-duration energy storage [11], and they play an important role in the energy transition and carbon neutrality paths. Hydrogen energy storage can be flexibly dispatched through compression, liquefaction, or solid state methods [12], and can be combined with renewable energy water electrolysis to form a “Power-to-Gas-to-Power” (P2G2P) cycle, which can not only absorb excess green electricity and relieve grid pressure, but also achieve energy cycle through reverse power generation by hydrogen fuel cells [13,14]. Redox flow batteries are particularly suitable for microgrids, energy storage power stations, and renewable energy grid connection scenarios due to their advantages of high safety, long life, and modular expansion [15]. Among them, the VRFB has excellent system reliability and economic potential due to its reusable electrolyte, low self-discharge rate, and good chemical stability [16]. Many recent studies have also indicated that if hydrogen energy and redox flow batteries are operated in conjunction through a hybrid energy storage architecture, the efficiency and dispatch flexibility can be improved by energy management strategies, and the stability and resilience of the power grid in the face of intermittent renewable energy input can be significantly enhanced [17]. Real-time multi-parameter monitoring is essential for optimizing the performance of hydrogen/vanadium redox flow batteries (HVRFBs). These data can also be utilized to develop state-of-charge (SoC) estimation models. The microsensor developed in this study serves as a high-precision hardware platform to achieve this objective.

Based on the above context, Micro-Electro-Mechanical Systems (MEMS) technology has been widely used in renewable energy and microsensors in recent years. Previously, this R&D team used organic solvents (acetone, methanol, and isopropyl alcohol) to clean the PI substrates of microsensors. However, the major problem with this cleaning method is that organic solvents can pollute and impact the environment, posing environmental and safety challenges.

In fact, similar problems have existed in the semiconductor industry for a long time. Organic solvents such as isopropyl alcohol (IPA) are often used for wafer cleaning. The emission of volatile organic compounds (VOCs) not only causes air pollution but also requires additional solvent recovery or thermal oxidation treatment equipment, resulting in increased energy consumption and carbon emissions, and posing challenges to corporate operating costs and ESG evaluations [18]. Therefore, the development of VOC-free green cleaning technology can not only reduce the environmental load, but also conform to the development trend of “low-carbon process” in the semiconductor industry.

To address these challenges, this study innovatively used the environmentally friendly cleaning agent B, developed by Sea Energe, to clean PI substrates. The cleaning agent has excellent surface decontamination capabilities, effectively replacing traditional organic cleaning methods and improving process compatibility and the reliability of microstructures. To validate its application potential, this study used a flexible nine-in-one microsensor as a platform and embedded it in the hydrogen flow channel of a HVRFB to monitor multiple parameters in real time during charge and discharge.

This study will compare Sea Energe cleaning agent B with organic solvents (acetone, methanol, and isopropyl alcohol) which were used by this R&D team for cleaning the PI substrates of microsensors to work out the differences between the two kinds of cleaning agents in MEMS microsensor fabrication. Actual HVRFB operation data were used to validate the reliability and advantages of Sea Energe cleaning agent B in energy and microsensor applications. The results of this study not only demonstrate the practical value of Sea Energe cleaning agent B in the MEMS process but also provide an important basis for the introduction of green processes into the semiconductor and MEMS fields.

## 2. Research Method

This study developed and designed a nine-in-one multifunctional sensing platform based on the flexible eight-in-one microsensor previously developed by this team [19]. The microsensor can simultaneously measure nine key operating parameters including voltage, current, conductivity, temperature, humidity, flow, pressure, hydrogen concentration and pH, thereby meeting the needs for real-time monitoring of multiple physical quantities of hydrogen/vanadium batteries. In terms of process, in combination with the existing multifunctional sensor microprocess technology, this study introduced the cleaning process of Sea Energe cleaning agent B for the first time to analyze the performance differences from traditional organic solvent cleaning methods. Through this process-improvement strategy, a nine-in-one microsensor that is acid-resistant, miniaturized, and process-compatible was successfully realized. Finally, this study embedded the developed cleaning agent B in the hydrogen end flow channel inside the HVRFB for real-time microscopic sensing and operation diagnosis. It is verified that the Sea Energe cleaning agent B has the normal performance of maintaining the reliability of the microsensor, demonstrating its actual value in a new generation of green processes [20], which has the potential to be extended to energy sensing, MEMS processes, and semiconductor manufacturing fields.

### PI Film Cleaning

This study employed the Taguchi method (L9 orthogonal array, four factors at three levels) for the experimental design. The four main factors were cleaning agent concentration, temperature, ultrasonic cleaning time, and DI water rinsing time. Each factor was set at three levels to systematically evaluate the influence of these parameters on the substrates cleaning performance. First, the PI film is cleaned. If the substrate surface is not clean and smooth enough, it will affect the stability of subsequent processes and even lead to poor adhesion of the metal film structure. The PI film is cleaned in the organic solvents of acetone, methanol, and IPA in turn, and then cleaned using an ultrasonic cleaner (DC300H, DELTA, Delta Ultrasonic Co., Ltd. Taipei, Taiwan) for 3 min. The substrate is rinsed with deionized water (DI water) to remove any residual organic solvents from the substrate surface, and then the substrate surface is purged using a high-purity nitrogen gun. The substrate is then baked on a hotplate (YS-300S, YSC, YOTEC, Hsinchu, Taiwan) at 110 °C for 5 min.

**Figure 1:**
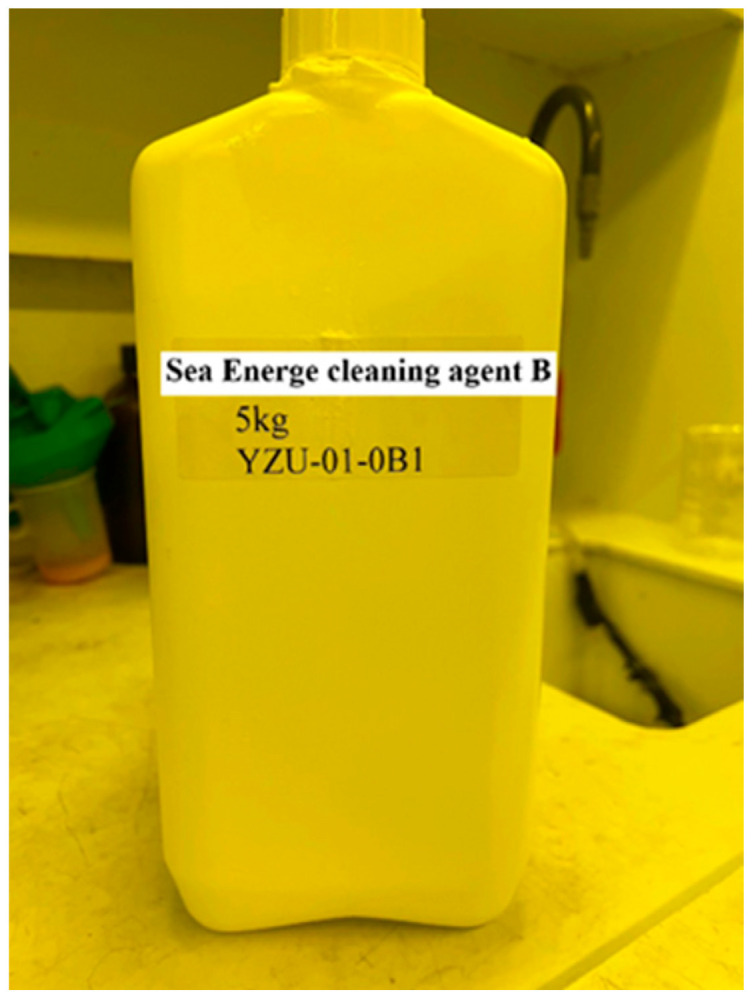
Sea Energe cleaning agent B.

**Figure 2:**
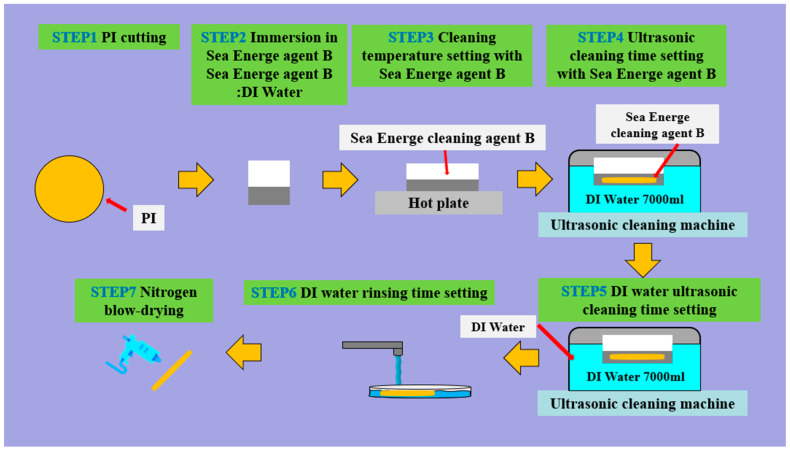
Substrate cleaning process in the microsensor process.

This study innovatively incorporates the Sea Energe cleaning agent B (Figure 1) into the substrate cleaning process in the microsensor process (Figure 2). This cleaning agent offers several advantages over traditional organic solvents (e.g., acetone, methanol, and IPA), including lower cost, no volatile organic gases, and no need for special wastewater recovery and treatment, resulting in good environmental friendliness and operational safety. Subsequently, the calibration and performance of microsensors fabricated using the samples treated with Sea Energe cleaning agent B and traditional solvents will be analyzed to evaluate the feasibility and practicability of the process.

## 3. Flexible Nine-in-One Microsensor Embedded in HVRFBs for Real-Time Microscopic Monitoring

**Figure 3:**
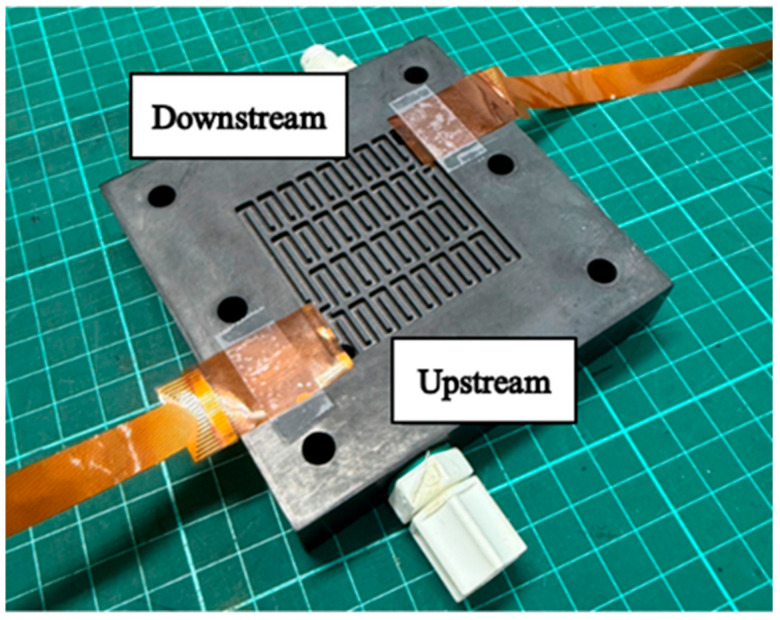
The upstream and downstream of the four serpentine non-parallel flow channels of the HVRFB, as shown in Figure.

**Figure 4:**
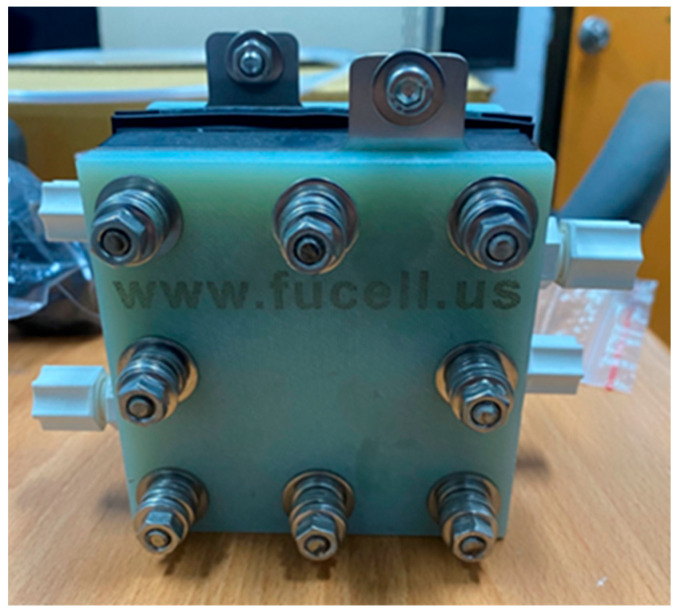
The HVRFB is shown in the figure.

According to the recent literature, the HVRFB used in this study employs standard graphite felt electrodes. It should be noted that surface modification of the electrodes (e.g., bismuth electrodeposition) can optimize reaction kinetics and consequently affect the signal stability of the microcurrent/voltage sensors, the nine key physical quantities (voltage, current, conductivity, temperature, humidity, flow, pressure, hydrogen, and pH) within a HVRFB are highly interactive, significantly affecting the overall performance and stability of the battery. To probe into the internal performance, this study embedded the flexible nine-in-one microsensors in the upstream and downstream of the four serpentine non-parallel flow channels of the HVRFB, as shown in Figure 3, and the sensors were coupled with a high-precision NI PXI-2575 data acquisition system to implement internal and local real-time microscopic monitoring of the battery. The HVRFB is shown in Figure 4.

## 4. Correction of Flexible Nine-in-One Microsensor

After the acid-resistant flexible nine-in-one microsensor was fabricated, it was corrected to confirm its proper operation before it was embedded in the HVRFB. This study will embed the flexible nine-in-one microsensor in the flow channel plate of the HVRFB to diagnose internal performance.

### 4.1. Correction Test for Micro Voltage, Current, and Conductivity Sensors

**Figure 5:**
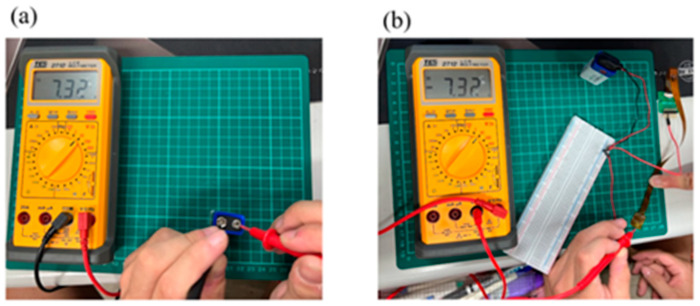
(**a**) Measured cell voltage; (**b**) battery measurement using a micro-voltage sensor.

**Figure 6:**
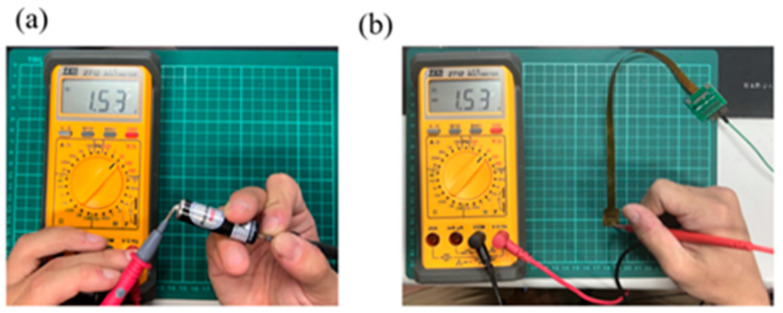
(**a**) Measured cell current; (**b**) battery measurement using a micro-current sensor.

A multimeter is used to check whether the measurement of micro-voltage sensor and micro-current sensor is correct. The correction procedure is that the battery voltage and current are verified by using the multimeter, and then the microsensor probe is connected to the battery to form a circuit, normal operation of the micro voltage, current, and conductivity sensors is verified if there is no error, as shown in Figure 5 and Figure 6. The voltage and current stability demonstrated by the sensor is partially attributed to the battery design. Future studies may consider integrating advanced separator technologies to further suppress ion crossover and enhance sensing consistency. The linear response exhibited by the microcurrent sensor indicates good electrode reaction efficiency. Further electrode modification (e.g., bismuth coating) could yield a smoother current output, offering potential value for high-precision sensing applications.

### 4.2. Correction Test for Micro-Temperature Sensor

**Figure 7:**
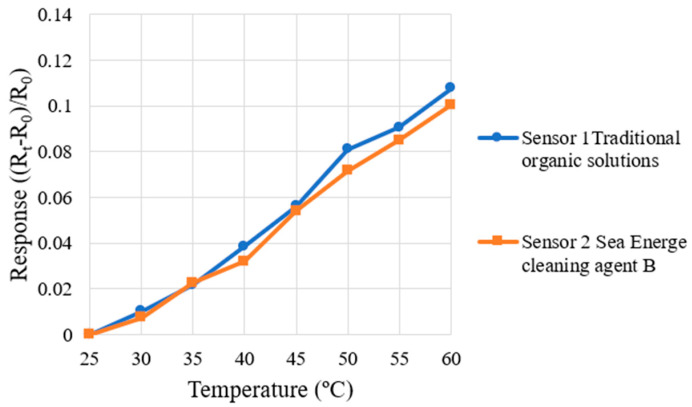
The correction curves of micro-temperature sensors are shown in the figure.

This study used a Programmable Temperature & Humidity Chamber (THS-A4T-150, KSON Co., Hsinchu, Taiwan) to correct the micro-temperature sensor. Given that the operating temperature of the HVRFB does not exceed 50 °C during actual operation, the temperature range was set as 25 °C to 60 °C for this correction test, with increments of 5 °C. The micro-temperature sensor was then placed in the chamber. When the temperature was stabilized, the resistance values (R_t_) at various temperatures were recorded using an NI PXI 2575 data acquisition system of National Instruments (NIs, Austin, Texas, USA) and compared with the resistance value (R_0_) at the initial temperature of 25 °C, and the dimensionless resistance variation ((R_t_ − R_0_)/R_0_) was calculated. Each micro-temperature sensor was measured three times, and the average value was taken to obtain the correction curve of each micro-temperature sensor. The correction curves of the micro-temperature sensors are shown in Figure 7. The micro-temperature sensor specifications are summarized in Table 1.

### 4.3. Correction Test for Micro-Humidity Sensor

**Figure 8:**
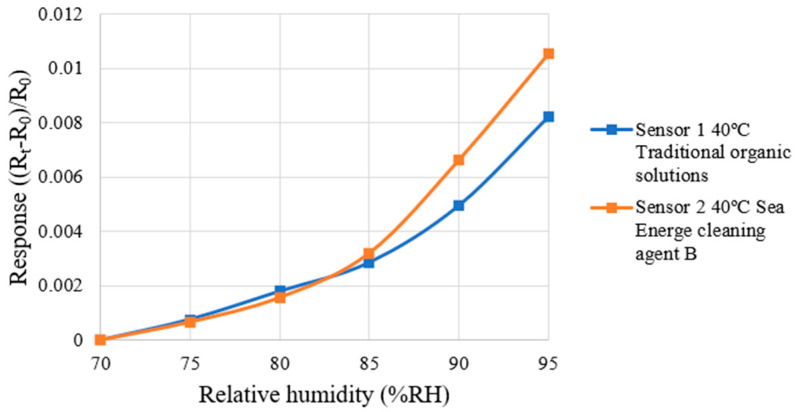
The correction curves of the micro-humidity sensors are shown in the figure.

This study used a programmable constant temperature and humidity tester to correct the micro-humidity sensor. The correction test was performed at a constant temperature of 40 °C, with relative humidity increased in 5%RH increments from 70%RH to 95%RH. To ensure the accuracy of the temperature and humidity values displayed by the constant temperature and humidity chamber, a commercially available temperature and humidity sensor was arranged as a reference during the correction test. Until the data of the constant temperature and humidity chamber and the commercial temperature and humidity sensor were consistent and stable, the sensing data in the time interval were recorded. After 30 min of humidity stabilization, the resistance value (R_t_) of the micro humidity sensor in the humidity range was recorded using the NI PXI-2575 data acquisition system and were compared with the resistance value (R_0_) at a relative humidity of 70%RH. The dimensionless resistance variation ((R_t_ − R_0_)/R_0_) was calculated. Each micro-humidity sensor was measured three times and the average value was taken to obtain the correction curve. The correction curves of the micro-humidity sensors are shown in Figure 8. The micro-humidity sensor specifications are summarized in Table 2.

### 4.4. Correction Test for Micro-Flow Sensor

**Figure 9:**
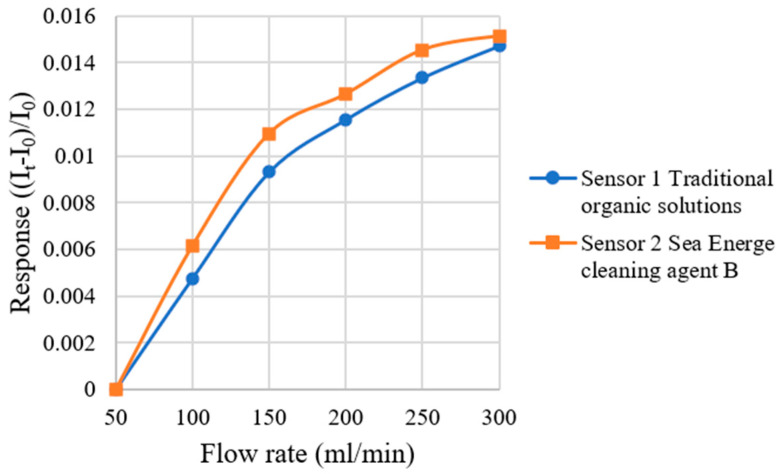
The hydrogen end correction results are shown in the figure.

For the correction test for the hydrogen end of the HVRFB, a fuel cell test system (850e, HEPHAS Co., Hsinchu, Taiwan) was used to provide a steady flow of hydrogen. The hydrogen temperature was 25 °C, and the flow range was set as 50 mL/min to 300 mL/min, measurements were performed at intervals of 100 mL/min. Under each flow condition, the current value (I_t_) of the micro flow sensor was captured using the NI PXI-2575 data acquisition system and compared with the current value (I_0_) at the initial flow of 50 mL/min, and the dimensionless current variation ((I_t_ − I_0_)/I_0_) was calculated. Each micro-flow sensor was measured three times and the average value was taken to obtain the calibration curve. The hydrogen end correction results are shown in Figure 9. The micro-flow sensor specifications are summarized in Table 3.

### 4.5. Correction Test for Micro-Pressure Sensor

**Figure 10:**
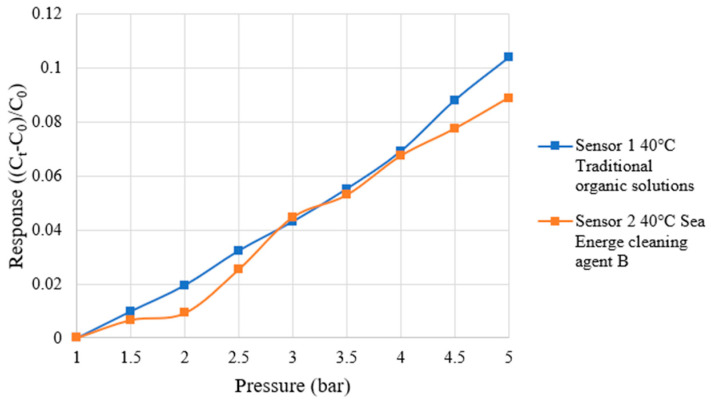
The correction curves of the micro-pressure sensors are shown in the figure.

During the correction of the micro pressure sensor, a hot press was used to apply a fixed pressure to the micro-pressure sensor. An impedance analyzer (LCR meter, 4230, Wayne Kerr Electronics Co., Chichester, UK) was used to capture capacitance data at various pressures and temperatures. To simulate the actual HVRFB operating environment, the temperature was set at 40 °C for the correction test, and 1~5 bar pressure was applied to the micro-pressure sensor at each temperature in increments of 0.5 bar. After each pressure condition was stabilized, the capacitance value (C_t_) of the micro pressure sensor at the current pressure was captured and compared with the capacitance value (C_0_) at the initial pressure of 1 bar. The dimensionless capacitance variation ((C_t_ − C_0_)/C_0_) was calculated. Each micro-pressure sensor was measured three times and the average value was taken to obtain the correction curve. The correction curves of the micro-pressure sensors are shown in Figure 10. The micro-pressure sensor specifications are shown in Table 4.

### 4.6. Correction Test for Micro Hydrogen Sensor

**Figure 11:**
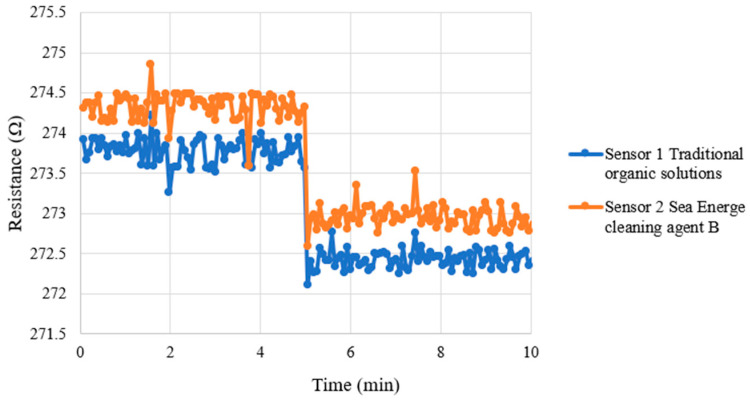
Micro hydrogen sensors show stable resistance shifts between O_2_ and H_2_, confirming reliable H_2_ response. Y-axis: (Ω_0_ − Ω), with Ω = current resistance and Ω_0_ = initial resistance.

For the correction of the micro hydrogen sensor, the high-purity hydrogen and oxygen provided by the fuel cell test system were used as test gases. The micro hydrogen sensor was installed on the flow channel of the HVRFB, the flow channel was used as a closed test environment. During the correction test, the temperature was set at 25 °C and a constant flow of oxygen was introduced, allowing the surface of the micro hydrogen sensor to adsorb oxygen ions. Afterwards, a constant flow of hydrogen was introduced. Under the same temperature conditions, hydrogen reacted with the oxygen ions on the surface of the micro hydrogen sensor and removed them, leading to a resistance drop of the micro hydrogen sensor. The resistance changes under different gas conditions were captured using the NI PXI 2575 data acquisition system to analyze the sensing changes in the micro hydrogen sensor. Figure 11 shows consistent resistance changes in two micro hydrogen sensors with switching between oxygen and hydrogen environments, meaning the micro hydrogen sensor has stable and consistent hydrogen response characteristics. The y-axis unit is (Ω_0_ − Ω), Ω represents current resistance and Ω_0_ represents the initial resistance.

### 4.7. Correction Test for Micro pH Sensor

**Figure 12:**
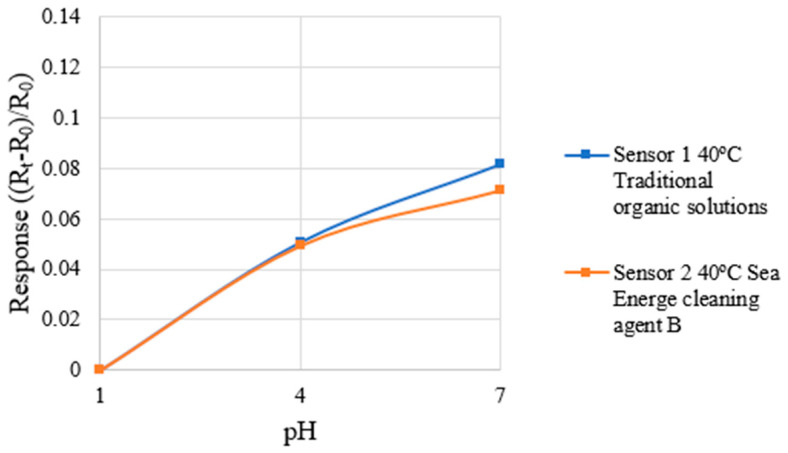
The correction results of the micro pH sensors are shown in the figure.

During the correction of the micro pH sensor, since the vanadium electrolyte used in the HVRFB was a strongly acidic solution, the correction test focused on the response behavior in the range of pH 1 to pH 7. A pH 7 standard solution was used as the initial reference solution for correction, and pH 4 and pH 1 acidic standard solutions were used as measuring solutions. The micro pH sensors were immersed in the standard solutions, respectively, and the corresponding resistance changes were measured. The pH-resistance correspondence was drawn to establish the correction curve of the micro pH sensor. The temperature was set at 40 °C for the correction test. To ensure measurement accuracy and stability of different solutions, the micro pH sensor was rinsed with DI water for 5 min after each correction and dried using a high-purity nitrogen gun to prevent measurement errors caused by residual solution. Each micro pH sensor was measured three times and the average value was taken to plot the correction curve. The correction results of the micro pH sensors are shown in Figure 12. The micro pH sensor specifications are summarized in Table 5.

## 5. Flexible Nine-in-One Microsensor Embedded in an HVRFB

### 5.1. Local Flow Distribution at Hydrogen End of HVRFB Embedded with Flexible Nine-in-One Microsensor

**Figure 13:**
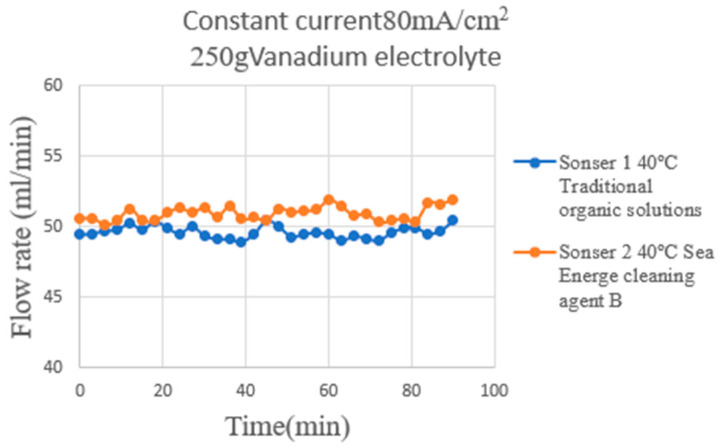
The hydrogen end of the HVRFB at the operating temperature of 40 °C during charge. The measurement results are shown in the figure.

**Figure 14:**
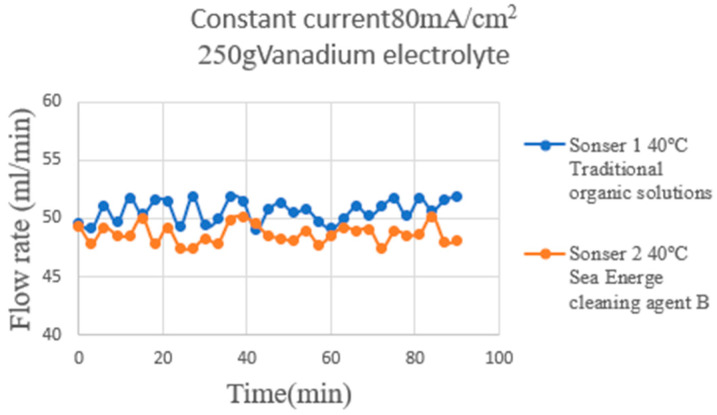
The hydrogen end of the HVRFB at the operating temperature of 40 °C discharge. The measurement results are shown in the figure.

This study also used the flexible nine-in-one microsensor to measure the flow changes at the hydrogen end of the HVRFB at the operating temperature of 40 °C during charge and discharge. The measurement results are shown in Figure 13 and Figure 14. The hydrogen supply flow was set at 50 mL/min. The measured results showed that the overall average flow was close to the set value, indicating stable hydrogen flow in the gas flow channel. Compared to liquid vanadium electrolyte, hydrogen has high compressibility and low viscosity, resulting in less flow resistance in the flow channel, thus preventing significant flow drops below the set value. The experimental results in this study were obtained as the mean ± standard deviation of three independent experiments.

During the charge period (Figure 13), a hydrogen evolution reaction occurs at the hydrogen end, releasing trace amounts of hydrogen from the electrode surface and accumulating in the downstream region of the flow channel. This phenomenon causes the flow measured by the downstream micro flow sensor to be slightly higher than that in the upstream. The average upstream–downstream flow difference is less than 1 mL/min, the overall flow is stable. This result demonstrates consistent hydrogen supply and evolution during the charging phase, and good flow-field distribution.

During the discharge period (Figure 14), hydrogen participates as a reactant in the electrochemical oxidation reaction and is continuously consumed in the negative electrode reaction zone. Although the overall average flow remains close to the set value, the hydrogen consumption induced by the reaction makes the downstream flow slightly lower than the upstream flow, and the flow curve fluctuates slightly. This variation is presumably due to the coupled behavior of the electrochemical reaction and gas transport. The overall variation is slight, with an average difference of less than 2 mL/min, remaining within a stable range, indicating that the hydrogen supply and flow field maintain good consistency during discharge.

### 5.2. Local Pressure Distribution of an HVRFB Embedded with Flexible Nine-in-One Microsensor

**Figure 15:**
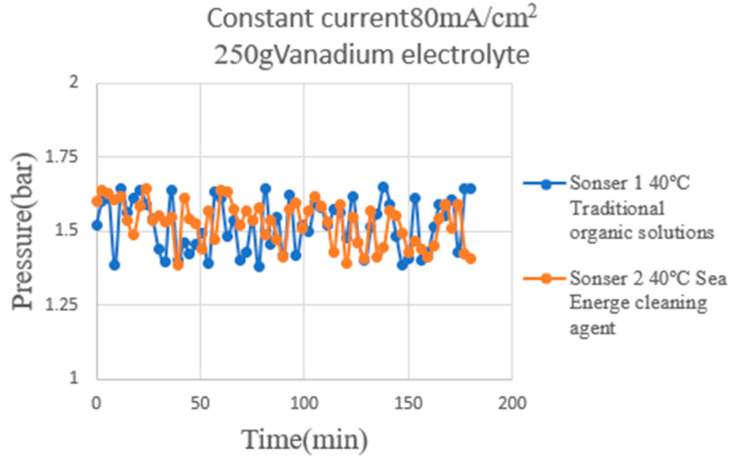
This study used the flexible nine-in-one microsensor to measure the internal pressure distribution at the vanadium electrolyte end and hydrogen end of the HVRFB during charge and discharge. Based on the measurement results, as shown in the figure.

This study used the flexible nine-in-one microsensor to measure the internal pressure distribution at the vanadium electrolyte end and hydrogen end of the HVRFB during charge and discharge. Based on the measurement results, as shown in Figure 15, the system’s flow-field stability and the airtight assembly quality are evaluated. At the vanadium electrolyte end (Figure 15), the pressure changes measured by the upstream and downstream micro-pressure sensors are stable, with no significant pressure drop or abnormal fluctuations, indicating smooth liquid flow in the flow channel, consistent pressure distribution, and no leakage. This is an ideal state, indicating good fluid circulation in the system and effectively preventing battery performance degradation due to pressure loss. The experimental results in this study were obtained as the mean ± standard deviation of three independent experiments.

### 5.3. Local Hydrogen Distribution of an HVRFB Embedded with Flexible Nine-in-One Microsensor

**Figure 16:**
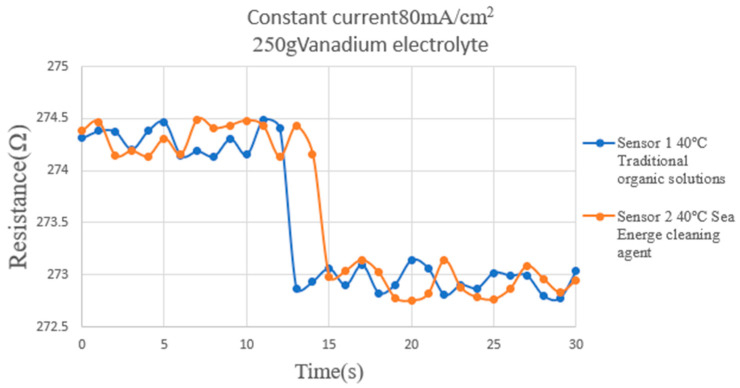
This study used a micro hydrogen sensor to measure the sensing response induced by hydrogen entering the flow channel of the HVRFB at 40 °C during the initial charging period. The results are shown in the figure.

This study used a micro hydrogen sensor to measure the sensing response induced by hydrogen entering the flow channel of the HVRFB at 40 °C during the initial charging period. The results are shown in Figure 16. The experimental design only explored the sensing changes caused by hydrogen entering the flow channel during the initial charging period. Figure 16 shows that when hydrogen enters the flow channel and contacts the micro hydrogen sensor, the resistance of the micro hydrogen sensor drops rapidly, indicating an immediate response. The experimental results in this study were obtained as the mean ± standard deviation of three independent experiments.

In addition, the initial resistance of the micro hydrogen sensor increases slightly with the operating temperature, a subtle and expectable difference. As hydrogen continues to enter and distribute throughout the flow channel, the resistance change in the micro hydrogen sensor gradually stabilizes.

## 6. Discussion

This study replaces the traditional organic solvent cleaning process with the Sea Energe cleaning agent B process. Traditional organic solvent cleaning requires the additional consideration of volatile solvents, while a single cleaning operation using Sea Energe cleaning agent B costs approximately TWD 14.48, which is significantly lower than the TWD 40 required for conventional organic solvent cleaning. This demonstrates its dual advantages of environmental friendliness and economic efficiency.

The proposed cleaning process has been successfully integrated into the MEMS fabrication flow. Compared with plasma cleaning, although plasma treatment provides excellent organic removal and surface activation capabilities, it still presents several limitations.

First, plasma cleaning relies on vacuum or atmospheric plasma systems and radio-frequency (RF) power supplies, leading to higher equipment investment and energy consumption costs [21]. Second, its cleaning performance is highly sensitive to parameters such as power, gas composition, pressure, and treatment time, resulting in poor reproducibility across different equipment [22]. In addition, for polyimide (PI) and other polymer substrates, excessive plasma power or prolonged exposure can cause increased surface roughness, bond breakage, or thermal damage, which in turn affects film adhesion and device stability [23].

In contrast, the Sea Energe cleaning agent can operate under ambient pressure and low energy conditions, with simple parameter control (concentration, temperature, and time). It exhibits excellent compatibility with PI substrates and does not require vacuum equipment, providing advantages in low cost, high safety, and green processing, making it highly suitable for flexible MEMS fabrication.

## 7. Conclusions

This study developed a flexible nine-in-one sensor based on the team’s multifunctional MEMS sensor process technology and introduced the cleaning process of Sea Energe cleaning agent B to compare it with traditional organic solvents. The results showed that Sea Energe cleaning agent B effectively removed substrate surface contamination and improved process compatibility, thereby ensuring the long-term stability of the microsensor in acidic electrolyte environments. The microsensor embedded in the hydrogen end of the HVRFB could simultaneously monitor nine parameters: voltage, current, conductivity, temperature, humidity, flow, pressure, hydrogen concentration, and pH, demonstrating good measurement accuracy and reproducibility. Further comparisons of the two processes revealed similar levels in sensitivity, sensing range, and accuracy, meaning that Sea Energe cleaning agent B can provide a more environmentally friendly alternative while maintaining measurement reliability.

Comprehensive comparison results show that the microsensors fabricated through the cleaning process of Sea Energe cleaning agent B not only demonstrate comparable or even superior operational stability to traditional organic cleaning methods, but also offer environmental advantages, low pollution levels, and high process compatibility. This result verifies that Sea Energe cleaning agent B can serve as a new generation of green cleaning solutions, with application potential in the fields of MEMS processes and semiconductor manufacturing. The data collected by the sensor can be further integrated into the SoC estimation framework to enable intelligent battery management, thereby maximizing the value of green processes in energy storage applications.

## Figures and Tables

**Table 1:** The micro-temperature sensor specifications are summarized in the table.

	Traditional Organic Solutions	Sea Energe Cleaning Agent
Sensing area	390 µm × 390 µm	390 µm × 390 µm
Sensitivity	0.00309 °C^−1^	0.00287 °C^−1^
Accuracy	±0.2 °C	±0.2 °C
Sensing range	25~60 °C	25~60 °C

**Table 2:** The micro-humidity sensor specifications are summarized in the table.

	Traditional Organic Solutions	Sea Energe Cleaning Agent
Sensing area	390 µm × 390 µm	390 µm × 390 µm
Sensitivity	0.00307%RH^−1^	0.00370%RH^−1^
Accuracy	±2%RH	±2%RH
Sensing range	70–95%RH	70–95%RH

**Table 3:** The micro-flow sensor specifications are summarized in the table.

	Traditional Organic Solutions	Sea Energe Cleaning Agent
Sensing area	390 µm × 430 µm	390 µm × 430 µm
Sensitivity (Liquid flow rate)	0.000308 mL/min^−1^	0.000395 mL/min^−1^
Accuracy (Liquid flow rate)	±2%mL/min	±2%mL/min
Sensing range (Liquid flow rate)	200~400 mL/min	200~400 mL/min
Sensitivity (Gas flow rate)	0.00388 mL/min^−1^	0.00417 mL/min^−1^
Accuracy (Gas flow rate)	±0.1 mL/min	±0.1 mL/min
Sensing range (Gas flow rate)	50~300 mL/min	50~300 mL/min

**Table 4:** The micro-pressure sensor specifications are shown in the table.

	Traditional Organic Solutions	Sea Energe Cleaning Agent
Sensing area	800 µm × 800 µm	800 µm × 800 µm
Sensitivity	0.0336 bar^−1^	0.0471 bar^−1^
Accuracy	±0.1 bar	±0.1 bar
Sensing range	1~5 bar	1~5 bar

**Table 5:** The micro pH sensor specifications are summarized in the table.

	Traditional Organic Solutions	Sea Energe Cleaning Agent
Sensing area	600 µm × 600 µm	600 µm × 600 µm
Sensitivity	0.0122 pH^−1^	0.0111 pH^−1^
Sensing range	pH 1~7	pH 1~7

## Data Availability

The original contributions presented in this study are included in the article. Further inquiries can be directed to the corresponding author.
